# Usefulness of Procalcitonin as a Predictor of Long-Term Prognosis in the Early Postoperative Period after Esophagectomy for Esophageal Cancer

**DOI:** 10.3390/jcm11123359

**Published:** 2022-06-11

**Authors:** Eisuke Booka, Hirotoshi Kikuchi, Ryoma Haneda, Wataru Soneda, Sanshiro Kawata, Tomohiro Murakami, Tomohiro Matsumoto, Yoshihiro Hiramatsu, Hiroya Takeuchi

**Affiliations:** 1 Department of Surgery, Hamamatsu University School of Medicine, 1-20-1 Handayama, Higashi-ku, Hamamatsu, Shizuoka 431-3192, Japan; booka@hama-med.ac.jp (E.B.); kikuchih@hama-med.ac.jp (H.K.); ryoma_irinomedical@yahoo.co.jp (R.H.); chestersnd@yahoo.co.jp (W.S.); sun_k_3324@yahoo.co.jp (S.K.); t.mura626@gmail.com (T.M.); matsumotomohiro1984@yahoo.co.jp (T.M.); hiramatu@hama-med.ac.jp (Y.H.); 2 Department of Perioperative Functioning Care and Support, Hamamatsu University School of Medicine, 1-20-1 Handayama, Higashi-ku, Hamamatsu, Shizuoka 431-3192, Japan

**Keywords:** esophageal cancer, esophagectomy, procalcitonin

## Abstract

The aim of this study was to investigate the relationship between serum procalcitonin (PCT) levels after esophagectomy and infectious complications and long-term prognosis. A total of 105 patients who underwent esophagectomy between 2012 and 2019 were stratified into two groups: PCT-High group of ≥1 ng/mL and PCT-Low group of <1 ng/mL. The clinical outcomes and prognostic factors were compared between the two groups 2 postoperative days (POD), 4 POD, and 7 POD after esophagectomy. As the postoperative days passed, the association between PCT and infectious complications became stronger, and the positive predictive value was 100% at 7 POD. At 2 POD, there was no significant association between PCT elevation and infectious complications. Patients in the PCT-Low group had significantly worse overall survival (OS) and recurrence-free survival (RFS) than those in the PCT-High group at 2 POD (*p* = 0.026 and *p* = 0.011, respectively). In multivariate analysis, advanced pathological stage (hazard ratio (HR), 5.348; 95% confidence interval (CI), 2.299–12.500; *p* < 0.001) and PCT-Low group at 2 POD (HR, 3.673; 95% CI, 1.116–12.092; *p* = 0.032) were also independent predictors of worse OS. PCT in the early postoperative period after esophagectomy could be a good predictor of prognosis.

## 1. Introduction

Esophageal squamous cell carcinoma (ESCC) has high malignant potential and poor prognosis, representing the sixth leading cause of cancer-related mortality worldwide, with more than 500,000 deaths in 2020 [1]. ESCC is an intractable cancer, although it can be expected to be completely cured at all stages except for distant metastatic cases. The combination of endoscopic treatment, surgical treatment, chemotherapy, and radiation therapy can be expected to improve the treatment results [2,3]. Esophagectomy is the mainstay for esophageal cancer treatment at all stages, but is more invasive than other treatments [4].

Serum procalcitonin (PCT) was firstly reported by Nylen et al., as a severe inflammatory marker based on the results of a study investigating patients with burns in 1992 [5]. PCT is a peptide consisting of 116 amino acids, with a molecular weight of about 13 kDa, and is normally synthesized in thyroid C cells as a precursor of calcitonin [6]. However, in serious infections caused by bacteria, parasites, and fungi, inflammatory cytokines such as TNF-α are produced by the action of cells and toxins [7]. In response to this stimulation, PCT is produced/synthesized by several organs such as the lungs, kidneys, liver, fat cells, and muscles, and is secreted into the blood [8].

PCT is a specific marker of bacterial infection and has been reported as a predictor of postoperative infectious complications after esophagectomy [9,10,11,12]. We hypothesized that PCT is a predictor of infectious complications after esophagectomy, and of long-term prognosis. In this study, we investigated the relationship between PCT levels after esophagectomy and infectious complications and long-term prognosis.

## 2. Patients and Methods

### 2.1. Patients

We retrospectively collected the data of consecutive patients with ESCC who were treated with esophagectomy at the Department of Surgery, Hamamatsu University School of Medicine between October 2012 and May 2019. All data were collected from the patients’ electronic medical records. All procedures were conducted according to the institutional and national standards on human experimentation and with the Declaration of Helsinki of 1964 and its later versions. The study was approved by the Ethics Committee of the Hamamatsu University School of Medicine (IRB No. 17–165). The board waived the requirement for written patient consent for the use of clinicopathological data, and all patients agreed to participate through an optout approach. All patients underwent esophagogastroduodenoscopy (EGD) and computed tomography (CT) from the neck to the pelvis to determine the clinical stage, which was diagnosed on the basis of the eighth edition of the Union for International Cancer Control tumor, node, metastasis classification scheme [13].

**Figure 1:**
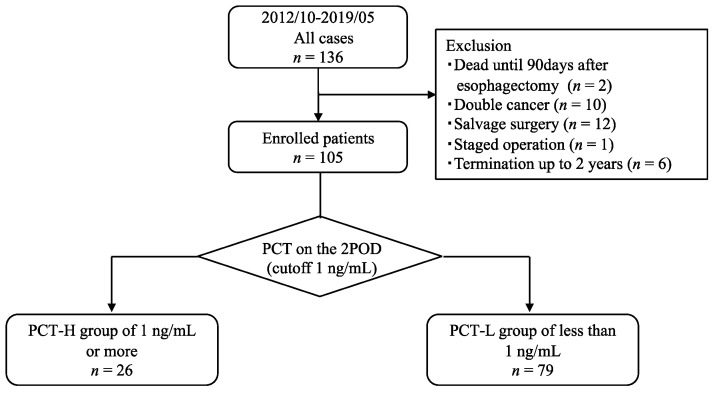
Study design diagram.

Patients who met the following criteria were enrolled in this study: (1) age > 20 years, (2) Eastern Cooperative Oncology Group performance status of 0 to 1, (3) histological diagnosis of ESCC by endoscopic biopsy, (4) no double cancer, (5) radical esophagectomy, (6) survival for at least 90 days after surgery, and (7) survived and were followed up for >2 years. Patient ineligibility for study enrolment was based on the following exclusion criteria: death within 90 days of surgery (*n* = 2), double cancer (*n* = 10), salvage surgery (*n* = 12), staged operations (*n* = 1), and interruption of follow-up within 2 years (*n* = 6). Finally, 105 patients were included in the study (Figure 1).

### 2.2. Blood Assessment for PCT and Determination of the Cutoff Value

Blood samples were collected directly in the morning of postoperative day (POD) 2, 4, and 7. PCT levels were measured using the Elecsys BRAHMS PCT assay (Roche Diagnostics GmbH, Mannheim, Germany) with an upper limit of 0.05 ng/mL. The cutoff value for the diagnosis of sepsis was 0.5 ng/mL, and the cutoff value for the severity of sepsis was 2.0 ng/mL. In this study, we set the cutoff value to 1.0 ng/mL, which is the third quartile in the second POD to investigate the association between PCT level and infectious complications after esophagectomy or long-term prognosis, with >1.0 ng/mL in the PCT-High group and <1.0 in the PCT-Low group.

### 2.3. Treatment and Postoperative Complications

Neoadjuvant chemotherapy was performed as a standard treatment for patients with non-Stage I ESCC. The treatment regimen was a combination of cisplatin and 5-fluorouracil or a combination of docetaxel, cisplatin, and 5-fluorouracil. Transthoracic esophagectomy with two- or three-field LN dissection and gastric conduit reconstruction via the posterior mediastinal route was performed as a standard surgical procedure at our institution [14]. In the thoracic approach, video-assisted or robot-assisted thoracoscopic surgery in the prone or the hybrid position was generally adopted [14]. Thoracotomy was performed for patients who refused thoracoscopy or were enrolled in another clinical trial [14]. Reconstruction using the right hemi-colon was performed when the stomach could not be used owing to a previous history of gastrectomy [15]. Postoperative complications were evaluated for pneumonia, anastomotic leakage (AL), and surgical site infection (SSI) using the Clavien–Dindo classification [16,17]. Complications of grade 2 or higher were identified as postoperative complications. Infectious complications included AL, pneumonia, SSI, pyothorax, and sepsis. Methylprednisolone was administered to the patients from 2 days before to 2 days after surgery.

### 2.4. Follow-Up

Postoperative follow-up was performed using CT every 6 months and EGD annually for 5 years after surgery [15]. Recurrence-free survival (RFS) was calculated from the time of surgery to the day of recurrence of esophageal cancer. Overall survival (OS) was calculated from the time of surgery to the day of death. Patients were followed up until death or until the end of the study (30 November 2021). Patients who interrupted follow-up or under follow were recognized as censored, and RFS and OS were calculated based on the days until censoring.

### 2.5. Statistical Analysis

The Statistical Package for the Social Sciences version 27.0 software (IBM Corp., Armonk, NY, USA) was used to conduct all statistical analyses. Categorical data were analyzed using Fisher’s exact test or the chi-square test where appropriate. Unpaired Student’s *t*-tests were used to analyze the quantitative data. A *p* value of <0.05 was considered statistically significant. Survival outcomes were analyzed using the Kaplan–Meier method and log-rank tests. Univariate and multivariate comparisons of survival time were performed based on Cox regression.

## 3. Results

### 3.1. Patient Characteristics

The characteristics of all study participants are shown in Table 1. The median age of the study population was 67 (range: 42–82) years, and most patients were male (87.6%). In almost half of the included patients, the tumor location was the middle esophagus (57.1%) and the cStage was I (45.7%).

The median PCT on 2 POD after esophagectomy was 0.35 (range: 0.05–8.57) ng/mL, and when 1 ng/mL was adopted as a cutoff and the patients were divided into two groups, 26 patients were in the PCT-High group (24.8%) and 79 patients were in the PCT-Low group (75.2%) at 2 POD. The clinicopathological features between the PCT-High group and PCT-Low group at 2 POD are shown in Table 1. There was no significant difference in the clinical stage between the PCT-High group and PCT-Low group (*p* = 0.140). There were also no significant differences in the following perioperative factors: thoracotomy, laparotomy, LN dissection, reconstructed organ, operation time, and blood loss. Multidisciplinary team support was significantly more common in the PCT-High group compared with the PCT-Low group (69.2% vs. 40.5%, *p* = 0.011). Postoperative infectious complications were not significantly different between the two groups, and PCT at 2 POD after esophagectomy was not associated with postoperative infectious complications. There were no significant differences in pStage or the proportion of postoperative adjuvant therapy between the two groups; however, the rate of regional LN recurrence was significantly lower in the PCT-High group compared with the PCT-Low group (7.7% vs. 30.4%, *p* = 0.020).

### 3.2. Relationship between PCT and Postoperative Infectious Complications at 2 POD, 4 POD, and 7 POD

The relationships between PCT and postoperative infectious complications at 2 POD, 4 POD, and 7 POD are shown in Table 2. Twelve of the 26 patients (46.2%) of the PCT-High group at 2 POD had infectious complications after 2 POD, and there was no association between PCT levels and infectious complications at this time. Infectious complications were observed in seven of nine cases (77.8%) in the PCT-High group at 4 POD, and PCT elevation was slightly related to infectious complications at 4 POD (*p* = 0.106). All six cases in the PCT-High group had infectious complications at 7 POD, and there was a significant correlation between PCT elevation and postoperative infectious complications after esophagectomy at 7 POD (*p* = 0.017). As the postoperative days passed, the association between PCT and infectious complications became stronger, and the positive predictive value was 100% at 7 POD. At 2 POD, there was no association between PCT elevation and infectious complications, and PCT elevation was considered a false positive associated with surgical invasion.

### 3.3. Patient Survival and Disease Recurrence

**Figure 2:**
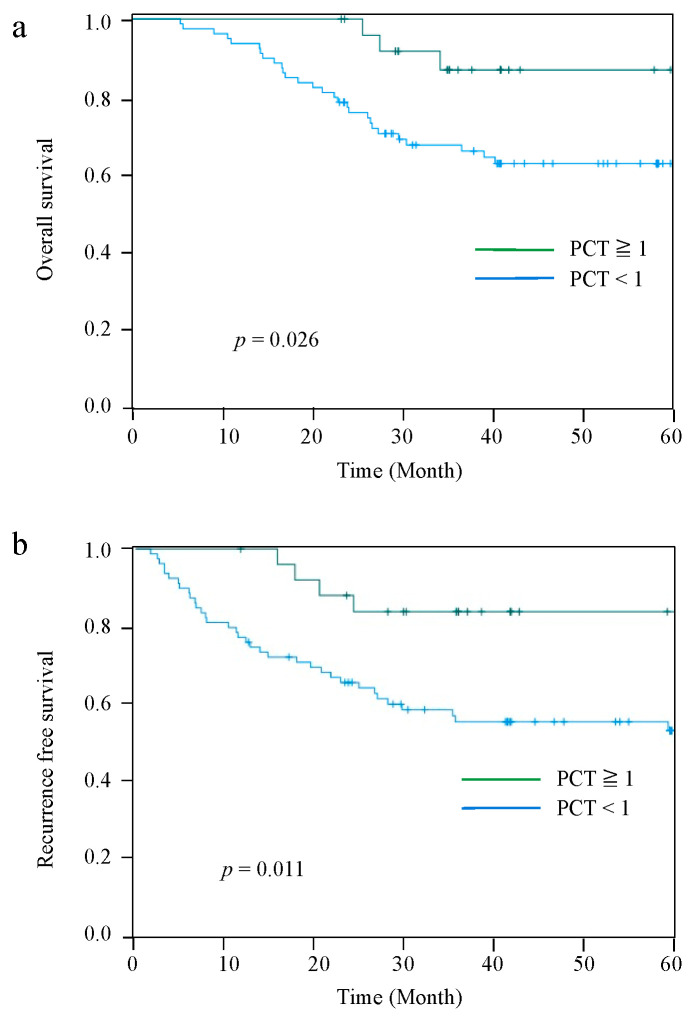
Kaplan–Meier curves for OS between the PCT-High group and PCT-Low group at 2 POD (**a**). RFS between the PCT-High group and PCT-Low group at 2 POD (**b**).

**Figure 3:**
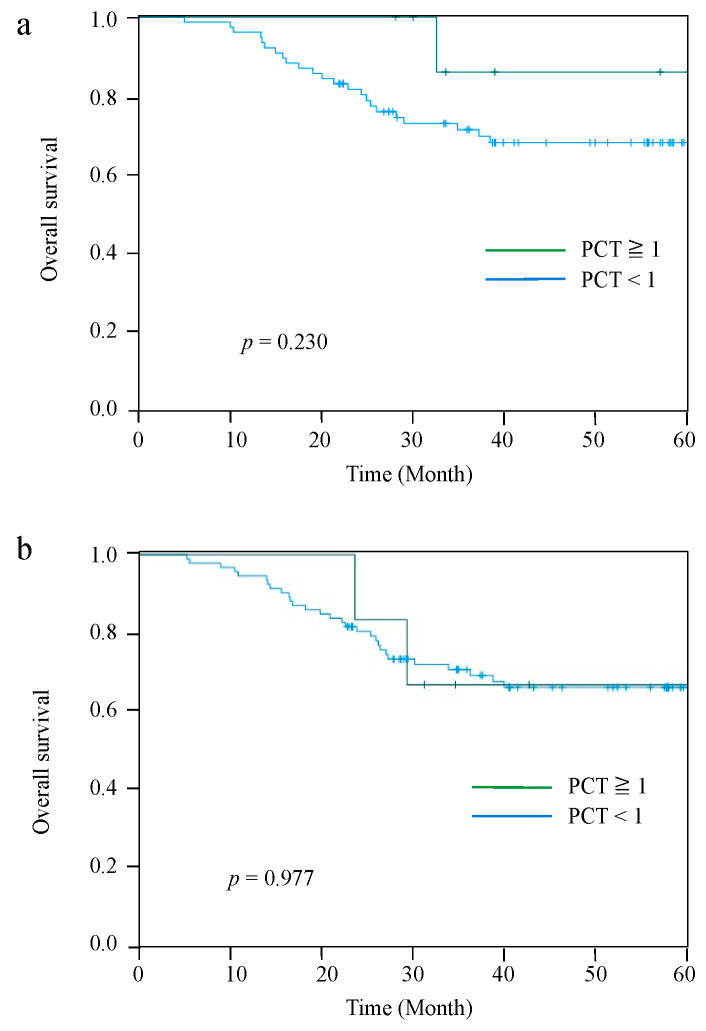
Kaplan–Meier curves for OS between the PCT-High group and PCT-Low group at 4 POD (**a**), and 7 POD (**b**).

The Kaplan–Meier curves for OS and RFS comparing the PCT-High and PCT-Low groups at 2 POD are shown in Figure 2. The median observation period for all cases was 42.5 (range: 5.6–84.5) months. Patients in the PCT-Low group had significantly worse OS and RFS than those in the PCT-High group (*p* = 0.026 and *p* = 0.011, respectively). The 5-year OS and RFS rates were 86.8% and 83.8%, respectively, in the PCT-High group, and 62.6% and 55.4%, respectively, in the PCT-Low group. However, there were no significant differences in OS and RFS between the PCT-two groups on 4 POD and 7 POD (Figure 3).

Table 3 shows the prognostic factors for patients who underwent esophagectomy. In univariate analysis, advanced pathological stage (≥II) and PCT-Low group at 2 POD were associated with a worse OS (*p* < 0.001, and *p* = 0.037, respectively). Postoperative complications (AL, pneumonia, and SSI) were not associated with OS. In multivariate analysis, advanced pathological stage (HR, 5.348; 95% CI, 2.299–12.500; *p* < 0.001) and PCT-Low group at 2 POD (HR, 3.673; 95% CI, 1.116–12.092; *p* = 0.032) were independent predictors of poorer OS.

## 4. Discussion

In this study, high PCT levels correlated with infectious complications at 4 POD and 7 POD, suggesting that PCT is a serum marker specific to infectious complications. In particular, the increase in PCT at 7 POD showed a 100% positive predictive value for infectious complications after esophagectomy. It has been reported that increased serum PCT after minimally invasive esophagectomy is associated with AL [9]. In cases where PCT is elevated at 7 POD, interventions such as antibiotic administration should be taken into consideration for infectious complications, including AL. However, it has been reported that the PCT becomes false positive at the time of severe invasiveness, such as in the case of severe trauma or in some diseases; however, no previous study has reported on the significance of the PCT false positive [6,18,19,20]. Bacterial toxins such as endotoxins act directly, and inflammatory mediators such as cytokines act indirectly, all of which are thought to enhance the elevation of PCT [21]. Therefore, it is considered that the PCT elevation immediately after esophagectomy was caused by inflammatory cytokines such as TNF-α, IL-1, and IL-6 [22].

We previously reported that the intensive postoperative inflammatory response after esophagectomy was significantly correlated with poor OS [23,24]. Surprisingly, in this study, high PCT levels in the early postoperative period were significantly correlated with good OS, suggesting that elevated PCT in the early postoperative period is not an inflammatory response, but a positive response for survival. We also previously reported that multidisciplinary team support was associated with a significant reduction in the incidence of postoperative pneumonia and significantly less weight loss [25]. The multidisciplinary team support included preoperative rehabilitation. In this study, multidisciplinary team support was significantly more common in the PCT-High group compared with the PCT-Low group at 2 POD. It is possible that preoperative team support boosts immunity preoperatively and increases the PCT level immediately after esophagectomy [26].

There are two possible reasons that an elevation in PCT without bacterial infection improves prognosis. First, PCT secreted into the blood induces monocyte migration and enhances the phagocytic ability of bacteria and, simultaneously, PCT activates T lymphocytes and promotes biological defense [18]. It has been reported that the expression of T lymphocytes after esophagectomy for ESCC reduces the recurrence of esophageal cancer and improves the prognosis [27]. In this study, both RFS and OS were better in the PCT-High group compared with those in the PCT-Low group at 2 POD, suggesting that the expression of PCT without bacterial infection induces T lymphocytes after esophagectomy and improves the prognosis.

Second, it is possible that antiserum reactive to PCT was produced in the PCT-High group at 2 POD. Indeed, previous animal studies have shown that administration of anti-PCT serum in a sepsis-induced model improves prognosis [18,28]. The anti-PCT serum, which was originally produced to reduce the severity of sepsis, may have suppressed the recurrence of esophageal cancer by being produced in a situation without bacterial infection. The rate of regional LN recurrence was significantly lower in the PCT-High group compared with the PCT-Low group at 2 POD (7.7% vs. 30.4%, *p* = 0.020). It is possible that anti-PCT serum suppressed regional LN recurrence after esophagectomy.

Recent studies have demonstrated the effectiveness of immune checkpoint inhibitors (ICIs) after esophagectomy for esophageal cancer as a postoperative adjuvant therapy [29]. The target selection is critical for postoperative adjuvant therapy, and the PCT-Low group could be the target of ICI administration after esophagectomy. The PCT-High group may have increased immunity owing to higher levels of T lymphocytes after esophagectomy, which would have prevented recurrence and improved prognosis by acting as an adjuvant ICI therapy, whereas the PCT-Low group might have not promoted immunity after esophagectomy [18]. Therefore, the administration of ICI to the PCT-Low group promoted immunity and could improve the prognosis after esophagectomy. PCT has been shown to be a specific serum marker for bacterial infection after 4 POD; however, PCT at 2 POD would be a predictor of prognosis after esophagectomy for esophageal cancer.

This study has some limitations that warrant discussion. The main limitation of our study is that the retrospective investigation was based on a small number of patients belonging to only one institution. Second, the measurement of PCT was performed at three points on the second, fourth, and seventh days after esophagectomy. An external validation study involving sufficient patients is needed to confirm our findings.

## 5. Conclusions

Esophagectomy is more invasive than other gastrointestinal surgeries and is associated with infectious complications [30]. PCT is a predictor of infectious complications after esophagectomy.

Our study demonstrated that high PCT levels in the early postoperative days after esophagectomy, as a first-line treatment for ESCC, are not statistically correlated to bacterial infections, being a good biomarker for prognosis.

## Figures and Tables

**Table 1:** Clinicopathological characteristics between PCT-High and PCT-Low at 2 POD.

	All Cases *n* = 105	PCT-High *n* = 26	PCT-Low *n* = 79	*p* Value
Age (median, years) †	67 (42–82)	66 (48–78)	67 (42–82)	0.992
Sex (%)				0.880
Male	92 (87.6%)	23 (88.5%)	69 (87.3%)	
Female	13 (12.4%)	3 (11.5%)	10 (12.7%)	
Preoperative body weight (median, kg) †	57.3 (36.0–82.0)	56.4 (41.6–78.3)	58.2 (36.0–82.0)	0.796
Preoperative BMI (median, kg/m^2^) †	21.2 (14.2–28.9)	21.3 (16.2–28.1)	21.1 (14.2–28.9)	0.749
Location of tumor (%)				0.162
Ut	11 (10.5%)	2 (7.7%)	9 (11.4%)	
Mt	60 (57.1%)	19 (73.1%)	41 (51.9%)	
Lt and Ae	34 (32.4%)	5 (19.2%)	29 (36.7%)	
Clinical Stage, TNM 8th (%)				0.140
Stage I	48 (45.7%)	11 (42.3%)	37 (46.8%)	
Stage II	26 (24.8%)	4 (15.4%)	22 (27.8%)	
Stage III	28 (26.7%)	11 (42.3%)	17 (21.5%)	
Stage IVA	3 (2.9%)	0 (0.0%)	3 (3.8%)	
Preoperative therapy (%)				0.952
None	56 (53.3%)	14 (53.8%)	42 (53.2%)	
NAC	49 (46.7%)	12 (46.2%)	37 (46.8%)	
Multidisciplinary team support (%)	50 (47.6%)	18 (69.2%)	32 (40.5%)	0.011
Thoracotomy (%)				0.776
Open	54 (51.4%)	14 (53.8%)	40 (50.6%)	
MIE	51 (48.6%)	12 (46.2%)	39 (49.4%)	
Laparotomy (%)				0.602
Open	45 (42.9%)	10 (38.5%)	35 (44.3%)	
Laparoscopy	60 (57.1%)	16 (61.5%)	44 (55.7%)	
LN dissection (%)				0.128
2-field	13 (12.4%)	1 (3.8%)	12 (15.2%)	
3-field	92 (87.6%)	25 (96.2%)	67 (84.8%)	
Reconstruct organ (%)				0.105
Gastric conduit	99 (94.3%)	26 (100%)	73 (92.4%)	
Colon conduit	6 (5.7%)	0 (0%)	6 (7.6%)	
Jejunostomy (%)	54 (51.4%)	10 (38.5%)	44 (55.7%)	0.127
Operation time (median, min) †	600 (318–1008)	628 (370–1008)	599 (318–982)	0.823
Blood loss (median, mL) †	345 (21–16,340)	407 (35–1670)	323 (21–16,340)	0.577
Complications, C–D grade, ≥2 (%)				
All infectious complications	53 (50.5%)	12 (46.2%)	41 (51.9%)	0.611
AL	16 (15.2%)	5 (19.2%)	11 (13.9%)	0.514
Pneumonia	31 (29.5%)	6 (23.1%)	25 (31.6%)	0.406
SSI	15 (14.3%)	4 (15.4%)	11 (13.9%)	0.854
Pathological stage, TNM 8th (%)				0.659
Stage 0	3 (2.9%)	0 (0%)	3 (3.8%)	
Stage IA/IB	33 (31.4%)	10 (38.5%)	23 (29.1%)	
Stage IIA/IIB	21 (20.0%)	5 (19.2%)	16 (20.3%)	
Stage IIIA/IIIB	34 (32.4%)	9 (34.6%)	25 (31.6%)	
Stage IVA/IVB	14 (13.3%)	2 (7.7%)	12 (15.2%)	
Adjuvant therapy (%)				0.127
None	59 (56.2%)	19 (73.1%)	40 (50.6%)	
Chemotherapy	45 (42.9%)	7 (26.9%)	38 (48.1%)	
Radiation	1 (1.0%)	0 (0%)	1 (1.3%)	
2 POD serum procalcitonin level (ng/mL) †	0.53 (0.05–8.57)	1.67 (1.00–8.57)	0.35 (0.05–0.93)	<0.001
Recurrence site * (%)				
Local recurrence	6 (5.7%)	1 (3.8%)	5 (6.3%)	0.636
Regional LN	26 (24.8%)	2 (7.7%)	24 (30.4%)	0.020
Distant organ	17 (16.2%)	3 (11.5%)	14 (17.7%)	0.458
Death unrelated to esophageal cancer (%)	3 (2.9%)	2 (7.7%)	1 (1.3%)	0.088

† Values are presented as median (range). * Some patients displayed multiple sites of recurrence. PCT: procalcitonin, BMI: body mass index, Ut: upper thoracic esophagus, Mt: middle thoracic esophagus, Lt: lower thoracic esophagus, Ae: abdominal esophagus, NAC: neoadjuvant chemotherapy, MIE: minimal invasive esophagectomy, LN: lymph node, C–D: Clavien–Dindo, AL: anastomotic leakage, SSI: surgical site infection, POD: postoperative day.

**Table 2:** The relationship between PCT level and infectious complications.

	All Cases	Infectious Complication (+)	Infectious Complication (−)	*p*-Value
2 POD serum procalcitonin level (ng/mL)	105	53	52	0.611
≥1	26 (24.8%)	12 (46.2%)	14 (53.8%)	
<1	79 (75.2%)	41 (51.9%)	38 (48.1%)	
4 POD serum procalcitonin level (ng/mL)	84	44	40	0.106
≥1	9 (10.7%)	7(77.8%)	2 (22.2%)	
<1	75 (89.3%)	37 (49.3%)	38 (50.7%)	
7 POD serum procalcitonin level (ng/mL)	98	52	46	0.017
≥1	6 (6.1%)	6 (100%)	0 (0%)	
<1	92 (93.9%)	46 (50.0%)	46 (50.0%)	

PCT, procalcitonin; POD, postoperative day.

**Table 3:** Independent factors of clinicopathological, surgical, and pathological features on shorter overall survival.

	Univariate Analysis			Multivariate Analysis		
	HR	*p*	95% CI	HR	*p*	95% CI
Age (years)	1.032	0.234	0.980–1.087			
Gender (Male vs. Female)	2.132	0.136	0.787–5.780			
Multidisciplinary team support (+ vs. −)	1.295	0.475	0.636–2.639			
Thoracotomy (Open vs. MIE)	1.12	0.754	0.552–2.273			
Laparotomy (Open vs. Laparoscopy)	1.063	0.735	0.746–1.514			
LN dissection (2-field vs. 3-field)	2.235	0.078	0.914–5.467			
All infectious complications (+ vs. −)	1.299	0.478	0.631–2.677			
AL (+ vs. −)	1.627	0.219	0.749–3.534			
Pneumonia (+ vs. −)	0.959	0.917	0.442–2.084			
SSI (+ vs. −)	1.756	0.122	0.860–3.584			
Pathological stage (≥II vs. <II)	5.263	<0.001	2.257–12.195	5.348	<0.001	2.299–12.500
2 POD serum procalcitonin level (<1 vs. ≥1)	3.553	0.037	1.080–11.695	3.673	0.032	1.116–12.092

HR, hazard ratio; *CI*, confidence interval; MIE, minimal invasive esophagectomy; AL, anastomotic leakage; SSI, surgical site infection; POD, postoperative day.

## Data Availability

The datasets generated and analyzed from this study are not publicly available to protect the anonymity of the participants but are available from the corresponding author, Hiroya Takeuchi, upon reasonable request.

## References

[1] Sung H., Ferlay J., Siegel R.L., Laversanne M., Soerjomataram I., Jemal A., Bray F. (2021). Global cancer statistics 2020: GLOBOCAN estimates of incidence and mortality worldwide for 36 cancers in 185 countries. CA Cancer J. Clin..

[2] Kitagawa Y., Uno T., Oyama T., Kato K., Kato H., Kawakubo H., Kawamura O., Kusano M., Kuwano H., Takeuchi H. (2019). Esophageal cancer practice guidelines 2017 edited by the Japan Esophageal Society: Part 1. Esophagus.

[3] Kitagawa Y., Uno T., Oyama T., Kato K., Kato H., Kawakubo H., Kawamura O., Kusano M., Kuwano H., Takeuchi H. (2019). Esophageal cancer practice guidelines 2017 edited by the Japan esophageal society: Part 2. Esophagus.

[4] Booka E., Takeuchi H., Suda K., Fukuda K., Nakamura R., Wada N., Kawakubo H., Kitagawa Y. (2018). Meta-analysis of the impact of postoperative complications on survival after oesophagectomy for cancer. BJS Open.

[5] Nylen E.S., O’Neill W., Jordan M.H., Snider R.H., Moore C.F., Lewis M., Silva O.L., Becker K.L. (1992). Serum procalcitonin as an index of inhalation injury in burns. Horm. Metab. Res..

[6] Becker K.L., Nylén E.S., White J.C., Müller B., Snider R.H.J. (2004). Clinical review 167: Procalcitonin and the calcitonin gene family of peptides in inflammation, infection, and sepsis: A journey from calcitonin back to its precursors. J. Clin. Endocrinol. Metab..

[7] Linscheid P., Seboek D., Nylen E.S., Langer I., Schlatter M., Becker K.L., Keller U., Müller B. (2003). In vitro and in vivo calcitonin I gene expression in parenchymal cells: A novel product of human adipose tissue. Endocrinology.

[8] Müller B., Peri G., Doni A., Perruchoud A.P., Landmann R., Pasqualini F., Mantovani A. (2002). High circulating levels of the IL-1 type II decoy receptor in critically ill patients with sepsis: Association of high decoy receptor levels with glucocorticoid administration. J. Leukoc. Biol..

[9] Asti E., Bonitta G., Melloni M., Tornese S., Milito P., Sironi A., Costa E., Bonavina L. (2018). Utility of C-reactive protein as predictive biomarker of anastomotic leak after minimally invasive esophagectomy. Langenbecks Arch. Surg./Dtsch. Ges. Fur Chir..

[10] Bogar L., Molnar Z., Tarsoly P., Kenyeres P., Marton S. (2006). Serum procalcitonin level and leukocyte antisedimentation rate as early predictors of respiratory dysfunction after oesophageal tumour resection. Crit. Care.

[11] Ito S., Sato N., Kojika M., Yaegashi Y., Suzuki Y., Suzuki K., Endo S. (2005). Serum procalcitonin levels are elevated in esophageal cancer patients with postoperative infectious complications. Eur. Surg. Research. Eur. Chir. Forschung. Rech. Chir. Eur..

[12] Li H., Wang D., Wei W., Ouyang L., Lou N. (2019). The predictive value of coefficient of PCT × BG for anastomotic leak in esophageal carcinoma patients with ARDS after esophagectomy. J. Intensive Care Med..

[13] Brierley J.D., Gospodarowicz M.K., Wittekind C. (2017). TNM Classification of Malignant Tumors.

[14] Booka E., Kikuchi H., Haneda R., Soneda W., Kawata S., Murakami T., Matsumoto T., Hiramatsu Y., Takeuchi H. (2021). Short-term outcomes of robot-assisted minimally invasive esophagectomy compared with thoracoscopic or transthoracic esophagectomy. Anticancer. Res..

[15] Haneda R., Hiramatsu Y., Kawata S., Honke J., Soneda W., Matsumoto T., Morita Y., Kikuchi H., Kamiya K., Takeuchi H. (2022). Survival impact of perioperative changes in prognostic nutritional index levels after esophagectomy. Esophagus.

[16] Clavien P.A., Barkun J., de Oliveira M.L., Vauthey J.N., Dindo D., Schulick R.D., de Santibañes E., Pekolj J., Slankamenac K., Bassi C. (2009). The Clavien-Dindo classification of surgical complications: Five-year experience. Ann. Surg..

[17] Dindo D., Demartines N., Clavien P.A. (2004). Classification of surgical complications: A new proposal with evaluation in a cohort of 6336 patients and results of a survey. Ann. Surg..

[18] Christ-Crain M., Müller B. (2005). Procalcitonin in bacterial infections—Hype, hope, more or less?. Swiss Med. Wkly..

[19] Carsin H., Assicot M., Feger F., Roy O., Pennacino I., Le Bever H., Ainaud P., Bohuon C. (1997). Evolution and significance of circulating procalcitonin levels compared with IL-6, TNF alpha and endotoxin levels early after thermal injury. Burns.

[20] von Heimburg D., Stieghorst W., Khorram-Sefat R., Pallua N. (1998). Procalcitonin—A sepsis parameter in severe burn injuries. Burns.

[21] Brunkhorst F.M., Heinz U., Forycki Z.F. (1998). Kinetics of procalcitonin in iatrogenic sepsis. Intensive Care Med..

[22] Assicot M., Gendrel D., Carsin H., Raymond J., Guilbaud J., Bohuon C. (1993). High serum procalcitonin concentrations in patients with sepsis and infection. Lancet.

[23] Matsuda S., Takeuchi H., Kawakubo H., Fukuda K., Nakamura R., Takahashi T., Wada N., Saikawa Y., Kitagawa Y. (2015). Correlation between intense postoperative inflammatory response and survival of esophageal cancer patients who underwent transthoracic esophagectomy. Ann. Surg. Oncol..

[24] Booka E., Kikuchi H., Hiramatsu Y., Takeuchi H. (2021). The impact of infectious complications after esophagectomy for esophageal cancer on cancer prognosis and treatment strategy. J. Clin. Med..

[25] Kawata S., Hiramatsu Y., Shirai Y., Watanabe K., Nagafusa T., Matsumoto T., Kikuchi H., Kamiya K., Takeuchi H. (2020). Multidisciplinary team management for prevention of pneumonia and long-term weight loss after esophagectomy: A single-center retrospective study. Esophagus.

[26] Zylstra J., Whyte G.P., Beckmann K., Pate J., Santaolalla A., Gervais-Andre L., Russell B., Maisey N., Waters J., Tham G. (2022). Exercise prehabilitation during neoadjuvant chemotherapy may enhance tumour regression in oesophageal cancer: Results from a prospective non-randomised trial. Br. J. Sports Med..

[27] Noma T., Makino T., Ohshima K., Sugimura K., Miyata H., Honma K., Yamashita K., Saito T., Tanaka K., Yamamoto K. (2021). Immunoscore signatures in surgical specimens and tumor-infiltrating lymphocytes in pretreatment biopsy predict treatment efficacy and survival in esophageal cancer. Ann. Surg..

[28] Nylen E.S., Whang K.T., Snider R.H.J., Steinwald P.M., White J.C., Becker K.L. (1998). Mortality is increased by procalcitonin and decreased by an antiserum reactive to procalcitonin in experimental sepsis. Crit. Care Med..

[29] Kelly R.J., Ajani J.A., Kuzdzal J., Zander T., Van Cutsem E., Piessen G., Mendez G., Feliciano J., Motoyama S., Lièvre A. (2021). Adjuvant Nivolumab in resected esophageal or gastroesophageal junction cancer. N. Engl. J. Med..

[30] Lazzarin G., Di Furia M., Romano L., Di Sibio A., Di Giacomo C., Lombardi L., Giuliani A., Schietroma M., Pessia B., Carlei F. (2020). Endoscopic Double-Pigtail Catheter (EDPC) internal drainage as first-line treatment of gastric leak: A case series during laparoscopic sleeve gastrectomy learning curve for morbid obesity. Minim. Invasive Surg..

